# Some Surgical Aspects of Carcinoma

**Published:** 1889-12

**Authors:** Nelson C. Dobson

**Affiliations:** Surgeon to the Bristol General Hospital; Lecturer on Surgery at the Bristol Medical School


THE BRISTOL

Medico-Chirurgical Journal.

DECEMBER, 1889.

### SOME SURGICAL ASPECTS OF CARCINOMA.

The Presidential Address, delivered Oct. 9th, 1889, at the opening of the 16th Session of the Bristol Medico-Chirurgical Society.

BY

Nelson C. Dobson, F.R.C.S. Eng.,

*Surgeon to the Bristol General Hospital;*
*Lecturer on Surgery at the Bristol Medical School.*

I feel that I owe my present position as President for the ensuing year of this Society to the fact that I am the last of those who took part in its original formation who has not already passed the Presidential Chair; and whilst I am pleased to occupy, by your kindness, the position of President, I am more pleased at the great success of the Society, whether looked at from the point of view of the scientific work done, or the number and enthusiasm of its members. When your retiring President and myself took a very active part in forming this

Society, we neither of us, I believe, thought it would become the important medical institution that it now is.

I have heard all the presidential addresses since the formation of this Society; and whilst I cannot hope to interest you as all my predecessors have done, and can say nothing that is new to you, I am, nevertheless, enabled to choose a subject for an address which has not yet been selected; viz., Some of the Surgical Aspects of Carcinomatous Diseases. In speaking of the surgical aspects, I use the term in a wide and liberal sense, including in my remarks many collateral questions, and do not limit it to those cases actually requiring operation; though the bent of my mind and the opportunities of my practice naturally turn in this direction.

I have from time to time, as the older members know, brought before your notice sarcoma in its various types: I shall, therefore, say little about it to-night; but direct your attention chiefly to the carcinomata—the abnormal structures, consisting of cells of an epithelial type variously grouped. And whilst drawing largely upon the labours of others with larger experience for ideas, suggestions, and facts, I shall endeavour to use the clinical material which has come before me for many of the observations which I make on the more practical points.

First of all, in considering the question, "Is cancer more common than it used to be?" I cannot, in answer, do better than refer to the *Morton Lecture on Cancer*, by Sir Spencer Wells. He there shows conclusively, by statistics, that it certainly is on the increase. If I were to judge from my own individual experience, I should say that in the last ten years it is vastly more common. But a limited experience like my own—or, indeed, any one man's experience is insufficient, however large his opportunities may be. I have no doubt, if a poll were taken of this meeting, that there would be a pretty general agreement in the opinion expressed by Sir Spencer Wells. This observer states, that in studying the reports of the Registrar General, the death-rate from cancer in 1884 was higher than in any previous year, and also that in three successive periods of ten years from 1851 to 1880, and in the seven following years to 1887, there had been a gradual increase of mortality from cancer,—an increase common to both sexes, but considerably greater in males than in females. I will not trouble you with statistics which are open to all of you, but will content myself by stating that from the year 1861 to 1887 there had been a steady increase in the deaths from cancer among one million persons living, as shown from the fact that the deaths in 1861 from cancer were 360 per million; in 1887 they had reached 606 per million. These facts speak for themselves, and cannot be accounted for by better diagnosis or more careful registration: they should stimulate every one of us to increased energy, and enquiry as to the cause of this great increase in a malady so terrible, and I am fain to confess, so little as yet amenable to treatment; an increase, too, which makes itself apparent in the later periods of middle-life—the period of greatest usefulness and responsibility.

It is stated also, on the authority of Dr. Fordyce Barker—whose acquaintance I had the pleasure of making in America two years ago, and who is known throughout the world as an eminent and careful physician,—that a like increase has been observed in the United States, and he specially mentions that in the city of New York the death-rate from cancer has risen from 400 to the million in 1875 to 530 in 1885. He also says,

"The disease is less common in the coloured than in the white races." This increase is a matter which concerns us all; and it is of national importance that a more systematic, classified, and comprehensive report should be issued by the Registrar General. It was, therefore, with extreme regret I read a statement made in the House of Commons, that at present such a task could not be undertaken on account of the cost that would be incurred. By such a report we might possibly learn that certain localities or trades were specially affected, and thus possibly gain some insight into the *cause* of the disorder. Certain it is that some cancerous affections appeared to have diminished in frequency—notably cancer of the scrotum, or chimney-sweep's cancer; and I have a belief that cancer of the lip is less common than it used to be, since the working-classes have pipes with wooden or bone mouthpieces: this is only conjecture and individual observation; I have no statistics to appeal to in support of the suggestion. Referring at this point to certain kinds of cancer, such as chimney-sweep's cancer, which are supposed to be due to special forms of irritation, as soot, &c., I may mention that, spending part of this summer's holiday in the Hebrides, I met Dr. Hector Cameron, the well-known Glasgow surgeon: and he mentioned to me a fact which he had noticed, as surgeon to the Western Infirmary; viz., that he and his colleagues met with large numbers of cases of cancer of the face, and that they nearly all occurred in Highland crofters; and he attributed this to the irritation produced by the peat-smoke and soot on the skin of the face. These crofters, for the most part, have going night and day peat fires, and the smoke and dust can only find exit by the door; this, coupled with a not too frequent application of soap and water, becomes a source of irritation similar to that produced in former days in this country among sweeps.

With regard to the geographical distribution of cancer, something has been done by the Collective Investigation Committee; but much more might have been done, had we each in our individual capacity taken the trouble to send in reports. These reports have not been sufficiently numerous to draw any thoroughly satisfactory general deductions from; yet some interesting points have been noticed in a summary by Mr. Butlin, published in the *British Medical Journal* of July, 1887. Thus, of the number of cancerous dwellers in town and country, it was found there were 136 in town and 70 in the country: but there are so many manifest circumstances to account for this disproportion, that it would not be safe from these returns to assume that people living in towns are more subject to cancer than those living in the country; whilst the return of those cancerous persons living in valleys and on hills was nearly equal.

In the character of the soil in which the dwellings stood there is a great difference. Without going into details, I may mention that the residences situated on the rock, the slate, the red sandstone, and the oolite, furnish an insignificant number; the chalk, a more considerable but yet small number; the gravel, a larger number; and the clays, by far the largest of all. There are other questions of residence, such as the proximity of a river or stream. These and kindred questions are of great interest to us, in our attempts to account for the greater frequency of cancer in certain localities.

The public also take some interest in the question of locality; and quite recently I was written to by a lady—a stranger to me—inquiring if Clifton and the neighbourhood occupied a favourable position on the "cancer-map." And, if not, if I could recommend her to some locality which had this enviable position.

There is another question with regard to cancer which has taken deep root in the public mind, and which influences us all, more or less, consciously or unconsciously, according to our bias in estimating the character of a doubtful case, and that is the question of heredity in cancer. I feel sure that much mental suffering, altogether unnecessary and uncalled for, is undergone by those who may have had one or other of their parents or grandparents, or some member of their family, affected by cancer, and who look upon every trivial pain or disorder as possibly cancerous because of their family history. Now, few of us would hesitate to affirm that a very large proportion of the cases of cancer that come under our notice show also other cases "in the family," but it may be brother or sister; in which case there could, of course, be no direct hereditary transmission in the strict sense of the word. And Sir James Paget has stated, as the result of his unrivalled experience, that in not more than one case in three, in his private practice, could hereditary influence be traced; and I am in the habit, and feel myself justified in it, in stating to patients with doubtful histories, and who not infrequently come to one fearing they have cancer on account of a supposed hereditary tendency,—I am in the habit, I say, of telling them that this question of heredity is by no means proven; and have thus been able to comfort some of them, a duty which is plain and obvious, if it can be conscientiously carried out—as, for my own part, I feel it can.

Of course, I do not underestimate the hereditary, any more than I underestimate the influence of blows as a producer of cancer. Few of us would deny that traumatism has an influence in the production of cancer, under certain circumstances and in certain conditions; but what those circumstances and conditions are we cannot at present determine; and in a similar manner I would argue with regard to heredity, only giving a stronger emphasis to heredity than to traumatism.

If we admit inheritance to be a predisposing cause, it is certainly not the only one. Probably there are many predisposing causes, and most certainly there are other causes at work in the production of cancer than inheritance.

Whatever be the causes of cancer—whether it be due to a microbe, as some affirm, and have even claimed to have discovered the particular bacillus which is characteristic of cancer,—I think we are justified in denying, for the present at least, the assertion of some, that the disorder is a contagious one. There is, so far as I know, no evidence of this. Quite recently, however, it has been shown to be inoculable.

Some years ago I made some experiments for the late Dr. William Budd, with the view of determining this question. I inoculated guinea-pigs, rabbits, and a dog, with cancer taken from an advanced cancer of the liver, simply inserting fragments under the skin. These experiments were followed by no appreciable result; and I stated to Dr. Budd at the time that there would be a better chance of success by using portions of cancer that had only recently been removed, and grafting after the method of M. Réverdin's skin-grafting, a method of treating granulating wounds which had just at that period been introduced. Quite recently, I noticed in the *Practitioner* for Nov., 1888, an account of cancer skingrafts: "Hahnn obtained the consent of a patient who had recurrent cancer of breast, and who had clearly only a few weeks to live, to transplant skin over the sound breast by pieces derived from skin over the affected breast. Numerous cancer nodules were cut off as evenly as possible on April 9th, and transplanted into ulcers formed by the removal of small portions of skin from the sound breast. On May 1st the transplanted skin had taken root, and ulcers were covered by epidermis. On May 19th, at edges of the pieces of skin some small projecting nodules appeared, the size of a millet seed; they gradually increased in size, and by June 26th they had reached the size of a cherry. Four days later the patient died; and the new growths were found to consist of well-developed connective tissue-stroma, with epithelial cells enclosed—typical microscopic characters of carcinoma. These masses had clearly insinuated themselves into healthy tissues, which were on all sides beginning to be invaded by epithelial nest.

This experiment is, so far as I know, the only one recorded of the successful transplantation of cancer. It is an experiment which cannot, and need not, be often repeated; but is important as showing the possibility of such inoculation during an operation.

Of course it may be argued, in the particular instance just recorded, that the patient was already cancerous, and therefore had a special predisposition to the development of cancer, and that the same would not occur in a perfectly healthy person. Whether this surmise be true or not, I venture to say none of us would think of testing the truth by experiment. But it has an important bearing also in making us increasingly careful of our selection of persons for ordinary skin-grafting cases, where the patient himself does not supply, as he mostly does, the necessary portions of skin. I have ventured to mention this, as I myself, some years ago, took an especial interest in this question.

Coming at length to the more practical point—viz., that of surgical interference in cancer,—we must start with some decided idea as to the local or constitutional origin of cancer; and I may say in the outset that I look upon cancer as at first a local disorder, capable of being cured by appropriate local means, and this idea is the basis of all my surgical work in connection with this frequent and terrible malady. I do not look upon a cancer nodule as being simply the local expression of a constitutional disorder, but as a local malady which, sooner or later,—and how soon nobody, in the present state of our knowlege, can say,—will, if left to itself, invade all or any tissue or organ of the body. My plea in doubtful cases is for early operation, and I urge it upon my medical brethren with all the power I possess. A wise and careful discrimination is necessary; but I say unhesitatingly, that if an error is to be made, let it be on the right side. It is far better that a doubtful growth should be early removed, which may finally turn out not to be cancer, than that a cancer should be allowed to wait until a diagnosis is certain, and when the patient is infected beyond the reach of cure. I speak this from a tolerably large experience, and I know it is the view of those most capable of judging. It may be said that this is a well-recognised fact among us. I can only say that if it is well recognised, "it is a custom more honoured in the breach, than the observance."

I have constantly to deplore too-late cases; and only quite recently, a lady who was brought to me with ulcerating scirrhus of the breast, which, for various reasons

I need not now discuss, I removed, afterwards told me that her former medical attendant, who had been trying to cure her for a year of the ulcer, assured her I had operated too early, and that the cancer was not "ripe" for removal.

From the cancers, especially of the breast, on which I have operated, and many others which I have seen in consultation, I am strongly convinced that we are not, as a profession, as yet sufficiently alive to the great importance of early operation, if anything like a cure (and I use the word cure with circumspection) is to be effected. I have many times had cases sent to me, both in hospital and private, which I have thought it my duty to decline to remove; and I am forced to the conclusion that, in the minds, at least, of a large number of the members of our profession, it appears that only fully developed and advanced cases are suitable for operation. Of course, I am not including those who for reasons of their own, which doubtless appear to them good and sufficient, advise their cancerous patients against operations: of these I have nothing to say, except that, as a general rule, I should not agree with them. What I desire to express as forcibly as I can is, that they shall not send these cases to the surgeon at the last moment, and so bring him (a matter of perhaps small concern), and the practice of surgery, (a matter of large and grave concern), into disrepute.

I know of my own experience of instances of cancer of breast, lip, and penis, on which I have operated, and in which there has been no recurrence for several years—that is to say, over six years,—and one instance of cancer of the breast more than fifteen years: of most of which cases I have microscopic specimens, and in which the diagnosis did not depend solely on my own judgment; so that there is no reason to doubt that the cases I name were genuine cases of cancer, any more than there is reason to doubt the practical cure of these cases.

In operating on cases of carcinoma, I do so under two different circumstances. The first, in which I consider there is a prospect of curing the patient, or at least prolonging life; and the second, in which there is no prospect of cure, and perhaps only a faint one of prolonging life, but in which I consider the remainder of life may be made more comfortable by the removal of a painful or offensive disorder, in which the patient suffers from a foul and acrid discharge, and has the mental worry of this added to the physical suffering. In a large number of cases, however, which I see, I am obliged to discourage the idea of operation, either because the disorder occurs in elderly people, where the progress of the disease is slow and comparatively painless,—I have under my observation several such patients;—or where the disorder has involved extensively glands and neighbouring structures, and where the risk of operation far out-balances the chances of doing good.

It may be argued that if the patient has a mortal disorder, no risk is too great, and it matters little if the patient's death is expedited by the operation: but that is not the surgeon's view of the matter, and I confess for myself that the more I see of such cases, the less I am disposed to interfere; indeed, I have no doubt that, with the best possible intentions, in certain cases, that probably we cannot as yet quite rightly select, the operation has appeared to give an increased stimulus to the growth. I have especially noticed this in scirrhus of the breast in comparatively young women, and particularly in the infiltrating variety, where the skin is early and deeply involved, with discolouration, and a brawny look and feel, and where, in consequence of the widespread infection of the breast and skin, it is almost impossible to get beyond the borders of the disease: these cases, unless seen quite early, had better be left alone, as a speedy recurrence and rapid rate of progress is almost certain.

In estimating the length of time that a patient remains free from recurrence after operation, we have more than one factor to deal with; viz., the freedom and thoroughness of the operation. Thus we have varying degrees of malignancy in cancers, not only in those of different varieties, as scirrhus, encephaloid, and epithelioma, but also in cancers of the same kind, and affecting the same tissue or organ of the body. Thus we see extreme degrees of difference in pain, rate of growth, and secondary deposits, in various cancers of the breast: we may see a typical scirrhus in appearance and microscopic structure running an exceedingly rapid course, with speedy affection of glands and internal organs; whilst another, precisely similar in gross and microscopic structure, going on slowly and lasting for years without affecting neighbouring glands or other structures. We may have, as it were, a mild attack of cancer, as we may have a mild attack of scarlatina; although the term mild can only be used as a comparative term, as cancer, however slow, is pretty sure to lead always—or, at least, almost always—to a fatal issue if left alone.

With regard to the difference in malignancy of the various forms of carcinoma—that is to say, with regard to the rapidity with which the various forms have a tendency to kill the patient, and to give rise to the greatest local extension and distress—I should, I think, from my own observation, place epithelioma first in the list. We are all familiar with the rapid extension and early death of patients with epithelioma of the tongue for instance: but I have noticed the same rapidity in local extension in epitheliomas of the male and female genitals, and occasionally in the lip; and whilst I look upon cases of early and free removal of epithelial cancers affecting the cutaneous and muco-cutaneous surfaces as amongst the most favourable and hopeful cases, I should be disposed to reverse this opinion in similar cases where the glands are already involved, although they may be freely removed at the same operation: some of the most extensive and hopeless recurrences have occurred to me in such cases as these, and especially have I noticed it in the lip and vulva. But probably the worst case I ever saw of local carcinomatous infection occurred in a well-known man, whom I was asked to see, suffering from phimosis and erysipelas, the latter affecting penis and neighbouring glands, and extending around the left side as far as the lumbar region, the red outline of the inflamed lymphatics being clearly mapped out nearly to the spine. The erysipelatous inflammation subsided after two or three weeks, during which I opened local abscesses, and as soon as possible slit up the foreskin, and found an epithelial sore, as I expected I should, under it. I removed the penis, and the patient returned to the country; but within three months there was cancerous deposit all along the inflamed area as far as the spine, and he speedily died of the widespread local trouble. It appeared as if the cancer infection had been carried along with the septic material which produced the erysipelas, and its morbific influence seems to have been intensified by the local inflammations in the same tract. This case in my experience is without parallel in its severity.

It was a singular coincidence that the wife of this patient suffered from scirrhus of the breast at the same time, and died of deposits in her liver just before her husband, having kept her own disorder secret until a few weeks prior to her death. It is a coïncidence, I think, without pathological importance.

It will be observed that I am speaking solely of surface carcinomas, or such affecting external parts as the breast or either extremity of the alimentary canal. I know little of the removal of cancers affecting the deeper parts of the alimentary tract, as of pylorus or colon, though I once removed a large epithelioma of the descending colon, which had become intussuscepted through the anus, with the result that the patient died in about thirty-six hours; but knowing the difficulty in early diagnosis of malignant growths which can be seen and felt, and also the liability to recurrence in those cases where one can get tolerably wide of the growth, I at present am not favourable to operations such as removal of pylorus or colectomy for malignant disease, and especially when the death-rate of the actual operation is so high as the best statistics at our disposal prove them to be. Somewhere, I believe, about 40 per cent. of the patients have died from the immediate effect of the operations; and when it is considered that this risk is incurred for what, at best, is only a brief respite, I am of opinion that we are not justified in it, notwithstanding that Billroth heralded the operation of removal of the pylorus as a new epoch in surgery. I am not fond of operations which are attended with considerable risk, and which, if successful, promise but a doubtful gain. I, however, find myself frequently doing operations which I formerly thought not justifiable, and am content to follow cautiously the more aggressive school of surgery.

In looking over my notes, I find that I have a brief record of 100 cases of carcinomas affecting the breast or cutaneous surfaces, or such as were readily accessible to operation. These cases are those on which I have operated and have notes: but many cases, I have no doubt, have not been recorded, such as cancers of lip, because I thought them (before I was specially interested in this subject) too trivial. These cases include 63 of breast; 2 of removal of upper jaw (in one of these cases I removed nearly the whole of both superior maxillæ simultaneously); 2 of lower jaw and 1 of palate; 6 of the tongue, 5 being in men and 1 in a woman; 11 of the lower lip, all men, no case occurring in the upper lip; 2 of the cheek; 5 of the penis; 2 of the vulva; and examples of epithelioma of temple, scalp, anus, leg, and sole of foot. I have, of course, seen many examples of malignant disease, in which I either did not think it right to operate, or in which I have only been consulted as to the propriety of operation; and as I have just spoken of the rapidity of recurrence in epitheliomas when the glands were already affected, I have some remarkable instances of the contrary—of slow progress in genuine epitheliomas of old people: one such case has been under my observation for twelve years; and as the patient is now in her eighty-third year, there is a fair prospect that she will not ultimately die of the disorder, though it may be considered as an accessory to the end when it does come. I have had a fair number of operations on the breast: I note 63 in all; but in looking further into them, I find 15 were adenomas, and 4 purely cystic, and 2 cystic sarcomas, one of the latter of which weighed 9 lbs.

There remain, therefore, 42 cases of cancer of the breast, and all, with one exception, have been cases of scirrhus; and, I think, of nearly all I have microscopic specimens. It is difficult to trace the final result of these cases, as many of them were in hospital practice; those of my private practice I know most about, and it is amongst these that I have chiefly to rely for cases remaining free for many years after operation. I can, however, say of the 63 operations that they all recovered from the operation, and in all the wound healed. In all the cases which I have called adenoma, the tumour simply was removed; and in none has there been a recurrence, though in one case a second tumour occurred in the same breast, and another tumour in the opposite breast, both small in size and simple in character. In the carcinomas, the whole breast was removed with two exceptions, in which, from the position of the growth, I was able to get very wide of it without removing the whole of the breast. In several of my later cases, but not as a matter of routine, I have cleared the axilla: all were done with antiseptic precautions, and without a single death. In one case I amputated both breasts at different periods, the last more than five years ago. The nature of the growth in this case was not typically scirrhus: the patient is still well.

It is difficult to trace hospital patients for years after an operation; but of my private cases, which some years ago were rarer than now, I have a patient who was alive and well a year ago. This patient had the breast only removed in 1874; and a few months later a recurrence in cicatrix, which was also removed. Another case remained well more than six years, and died, I believe, of cancer of tongue. Another patient is alive and well after seven years:

in this case I had, two years after removing the breast, to remove a small tumour from the soft palate, which proved to be a round-celled sarcoma. This breast was a typical scirrhus, and was seen by Sir James Paget. Another patient had breast removed and axilla cleared in 1884, and is still well: there was in this case a particularly strong family-history of cancer. I have, of course, others alive after shorter periods; those I have named are a few well-marked instances of freedom from recurrence for several years, and occurring in the comparatively small number of cases I have had in my private practice. I am familiar with the statistics of Banks and Gross, and I do not consider they justify such severe operations as they advocate: they both show a larger mortality from operations than is compensated for, in my opinion, by subsequent immunity for long periods. These operators have done good service by insisting on free and wide removal; but, in my opinion, they go too far in advocating complete slicing off of the breast, and clearing the axilla in all cases. There is still a difference of opinion as to whether the entire breast should be removed in every case. As a rule, no doubt it should; but I quite agree with Butlin that now and then we meet with cases, especially when the growth is situated on the margin of the breast, where this is not necessary. I have practised this free removal of growth, but not entire removal of breast, in two cases within the last two and a half years: in the one case there is recurrence, not local; but in the other, done twelve months ago, the patient remains well. I have often seen the incisions coming quite close to the growth, and needlessly far from the growth at a lower point; and I have no doubt some of these cases would have remained longer free if a wide removal of the growth only had been practised. I do not wish to be misunderstood. I believe generally in carcinoma of the breast it is better to remove the whole breast; but I believe that occasionally we meet with cases in which the growth may be freely removed without removing the whole breast; and in such cases I now think that free removal, with extirpation of the whole breast, would be a needlessly severe operation. I know that this is contrary to the general opinion, and directly contrary to the opinion recently expressed by Sir Spencer Wells; but I believe it is an opinion held by a growing number of surgeons who are well able to judge of its merits. I have had but a very limited experience of the treatment of cancers of the breast by any other operative measures than removal by the knife; but I have occasionally used caustics, and I think in proper cases this method may sometimes be useful: and I have no knowledge of the arrest or cure of cancer by powerful interrupted Voltaic currents, as advocated by Inglis Parsons quite recently.

In addressing the Bristol Medico-Chirurgical Society, I am aware that I am addressing a body not exclusively surgical, and I trust my more purely medical friends will excuse me if the subject I have chosen is not of interest to them: but a disorder such as that under consideration is of interest to us all alike, and I, as a surgeon, look to my medical friends for help and guidance; and whilst I at present think early and free operation the only chance of cure in suitable cases of cancer, I look forward to the time when this dire disease will be as much under the control of medicines as is syphilis at the present day. What the particular remedy or remedies may be, is a question of the future: but I fear we cannot at present rely on any known medicine for the cure of cancer; certain it is that we have not yet found it in either Chian turpentine, or papaine and thallin, combined or not with vegetable diet, or any other drug as ordinarily administered. Familiar as I am with the enterprise of the more strictly medical members of this flourishing Society, it is not too much to hope that something may yet be done amongst them in furtherance of such a desirable object; none would more gladly welcome such assistance than myself. Meanwhile, it appears to me that in superficial cancers we have, up to the present, no remedy so reliable as early and free removal; and I end what I have to say by a plea for early and exact diagnosis, and early reference to the surgeon.

